# Incidence of H1N1 2009 Virus Infection through the Analysis of Paired Plasma Specimens among Blood Donors, France

**DOI:** 10.1371/journal.pone.0033056

**Published:** 2012-03-22

**Authors:** Angie Bone, Jean-Paul Guthmann, Azzedine Assal, Dominique Rousset, Armelle Degeorges, Pascal Morel, Martine Valette, Vincent Enouf, Eric Jacquot, Bertrand Pelletier, Yann Le Strat, Josiane Pillonel, Laure Fonteneau, Sylvie van der Werf, Bruno Lina, Pierre Tiberghien, Daniel Lévy-Bruhl

**Affiliations:** 1 Institut de Veille Sanitaire, St Maurice, France; 2 European Programme for Intervention Epidemiology (EPIET), European Centre for Disease Control and Prevention, Stockholm, Sweden; 3 Etablissement Français du Sang, La Plaine Saint Denis, France; 4 Institut Pasteur, Unit of Molecular Genetics of RNA viruses, National Influenza Center (Northern-France), Department of Virology, Paris, France; 5 Centre national de référence du virus influenzae (région Sud), Lyon, Groupement Hospitalier Est, Hospices Civils de Lyon & EMR Virpath, Université Claude Bernard Lyon1, Université de Lyon, Lyon, France; 6 Centre National de la Recherche Scientifique, Unité de recherche associée 3015, Paris, France; 7 Université Paris Diderot, Sorbonne Paris Cité, Paris, France; University of Hong Kong, Hong Kong

## Abstract

**Background:**

Knowledge of the age-specific prevalence of seroprotection and incidence of seroconversion infection is necessary to complement clinical surveillance data and statistical models. It provides the basis for estimating the future impact of influenza A (H1N1pdm09) and implementing appropriate prevention and response strategies.

**Methods:**

Using a cross-sectional design, two-stage stratified sampling and paired plasma samples, we estimated the age-specific prevalence of a protective level of H1N1pdm09 antibodies in the French adult population before and after the 2009/10 pandemic, and the proportion of those susceptible that seroconverted due to infection, from a single sample of 1,936 blood donors aged 20–70 years in mainland France in June 2010. Samples with a haemagglutination inhibition (HI) titre ≥1∶40 were considered seropositive, and seroconversion due to infection was defined as a 4-fold increase in titre in the absence of H1N1pdm09 vaccination or pre-pandemic seropositivity.

**Results:**

Out of the 1,936 donors, 1,708 were included in the analysis. Seroprevalence before the pandemic was 6.7% (95% CI 5.0, 8.9) with no significant differences by age-group (p = 0.3). Seroprevalence afterwards was 23.0% (95% CI 17.7, 29.3) with 20–29 year olds having a higher level than older groups (p<0.001). Seroconversion due to infection was 12.2% (95% CI 6.9, 20.5). Younger age-group, vaccination against H1N1 and being seropositive before the pandemic were strongly associated with post-pandemic seropositivity.

**Conclusions:**

Before the 2009/2010 winter influenza season, only 6.7% of the French mainland population aged 20–70 had a level of antibodies usually considered protective. During the first pandemic wave, 12.2% of the population seroconverted due to infection and the seroprevalence after the wave rose to 23%, either due to prepandemic seropositivity, infection or vaccination. This relatively low latter figure contributed to an extension of target groups for influenza vaccination for the 2010/2011 season.

## Introduction

The pandemic wave of influenza A (H1N1) 2009 occurred in France over 16 weeks (October 2009–January 2010) [1]. Between 8–14.8 million people were estimated to have been infected in mainland France, from clinical surveillance data adjusted for estimated proportions of asymptomatic cases and symptomatic cases not reporting to health services [2]. Almost 5.2 million were vaccinated against the pandemic A(H1N1)2009 (H1N1pdm09) virus in a national vaccination campaign launched in November 2009, resulting in an uptake of 8% [3].

Knowledge of the prevalence of immunity after a pandemic wave is necessary in order to estimate the future burden of disease and to plan appropriate response strategies. Information on the prevalence of immunity prior to the pandemic and the proportion of the population seroconverting contributes to our understanding of the epidemiology of the infection. Estimates of these measures can be derived by modelling using clinical surveillance data, but the limitations of these approaches are well recognized [4]. Direct measurement of antibodies to H1N1pdm09 through serological methods enhances these estimates.

Several cross-sectional seroepidemiological studies in a variety of populations before or after the pandemic wave(s) have been published [5]. Few have been able to obtain serial samples from the same individuals [6]–[8], and thus able to directly assess the proportion of subjects seroconverting, or the impact of a protective level of cross-reactive antibodies before the onset of the pandemic on subsequent seroprevalence.

We report the results of a national serological study in mainland France carried out in blood donors, thus enabling access to linked plasma samples taken before and after the pandemic wave in a given individual. Our first objective was to estimate the age-specific seroprevalence of a protective level of antibodies to H1N1pdm09 in adults before and after the 2009/10 pandemic wave. We also estimated the percentage of seroconversion that could be attributed to infection. Although we based our work on the analysis of plasma, the word « seroconversion » is used throughout the article.

## Methods

### Study design

We performed a cross-sectional study of blood donors aged 20–70 years donating during two weeks in mainland France in June 2010. We excluded donors who had not donated between January 2005 and April 2009. Donors were selected among the population of donors presenting at a blood collection site to donate their blood, without any screening or additional selection procedure. To ensure a random selection of these donors, we used a random stratified two-stage sampling design. The first stage involved unequal probability sampling of blood collection sites proportional to regular donor activity in June 2009, stratified by 14 mainland French blood service (Etablissement Français du Sang) regions and type of blood collection site (fixed, mobile urban, mobile rural). Mobile sites were designated urban if they were situated in urban units of more than 20,000 habitants using the National Institute of Statistics and Economic Studies (Insee) classification [9]. At the second stage, donors were randomly recruited at each selected blood collection site (two in each 10-year age group at fixed sites and one in each group at mobile sites). We aimed to achieve a sample size of 350 in each 10-year age-group based on a maximum seroprevalence of 30%, an alpha error of 5% and a precision of 5%. To allow for possible recruitment difficulties, blood collection sites were instructed to aim for a total of 420 donors in each age-group, or 2100 donors overall.

The study complied with the participating institutions guidelines for human research. According to French law, formal ethical committee clearance was not required for this study as donors provide written consent to the use of blood samples for research as part of standard blood donation procedures and all data collected for study purposes were anonymous. The French Commission for Data Protection (Commission Nationale de l'Informatique et des Libertés) gave approval for the study. On presenting to donate blood, donors were provided with a letter explaining the study, the provisions to ensure confidentiality, and the opportunity not to participate. Those who gave oral consent, documented by responding to our questionnaire, were included in the study until the target sample size for each age-group at that blood collection site was reached.

Participating donors were questioned about influenza-like symptoms (a respiratory infection with fever and cough and myalgia or fatigue) from October to December 2009, and vaccination against influenza H1N1pdm09. A sample of plasma was retained from the donation and a stored plasma sample prior to April 2009 was retrieved. Stored plasma samples are linked to a donor using a unique identification number. It has been mandatory in France to store plasma specimens from every blood donation for 5 years since 1999. At each donation, two plasma specimens (500 µL) are prepared (Cryo Bio Straw, Cryobiosystem, L'Aigle, France) and frozen in liquid nitrogen.

### Laboratory methods

Antibody titres against influenza A/California/7/2009 (H1N1) were measured by haemagglutination inhibition assays (HI) on all samples using standard techniques [10], [11]. To avoid misreadings of HI titers on plasma specimens, triple readings were carried out independently. Indeed, determination of HI titers in plasma can be difficult due to partial reactions. To overcome this problem, chicken red blood cells were selected, and additional readings of the HI plates were carried out after 1 hour at +4°C. If partial HI remained observed, the plasma was retested. Unpublished data have shown an excellent consistency of results when using chicken RBC rather than turkey RBC, which are not always readily available. Titres were expressed as the reciprocal of the highest dilution of serum where haemagglutination was inhibited. Microneutralization (MN) assays were performed on a random selection of 30% of sample pairs for comparison.

### Statistical analysis

Each donor was assigned a sampling weight based on the inverse of inclusion probabilities at each sampling stage. This enabled us to account for the complex sampling design so that donors with a greater chance of being selected had less weight than those with a smaller chance of selection. Sampling weights were further adjusted by age and sex using data from the 2008 French census (National Institute of Statistics and Economic Studies, Insee) in order to provide estimates for the mainland French population aged 20–70 (seeappendix S1). Age-specific geometric mean titres (GMT) were calculated by assigning a value of 5 for titres lower than 10 and 1280 for titres of 1280 or higher.

Samples with an HI titre of ≥1∶40 were considered seropositive, this being the titre conventionally used as it was shown to be associated with at least a 50% reduction in the risk of infection or disease with seasonal influenza viruses [12]. Seroconversion was defined as a 4-fold increase in HI titre between the two samples. For analyses estimating seroconversion due to infection, pairs with a titre of ≥1∶40 in the pre-pandemic sample (considered not susceptible) or with H1N1pdm09 vaccination (alternative explanation for seroconversion) were excluded. Prevalence of seropositivity before and after the 2009/10 pandemic and the proportion seroconverting was estimated by demographic factors and other exposure variables. Log-Poisson regression models with a robust linearized variance estimator were used to estimate adjusted prevalence ratios for seropositivity after the pandemic and seroconversion due to infection, since odds ratios calculated from logistic regression models are not a good approximation of the prevalence ratio when the prevalences in the exposed and unexposed groups are very different [13], [14] (see appendix S1). Variables were entered into the multivariable models and removed manually by stepwise selection, retaining those significantly associated with the outcomes at a significance level of p≤0.05. The triple interaction between age, preexisting seroprotection and A(H1N1)2009 vaccination status was included and tested for significance as it was anticipated that the likelihood of post pandemic seropositivity would differ between combinations of the various levels of those variables.

All data management and analyses were performed by the Institut de Veille Sanitaire team with STATA 11.2 [15], using the svy prefix to incorporate the sampling design (stratification, stages, weights).

## Results

**Figure 1:**
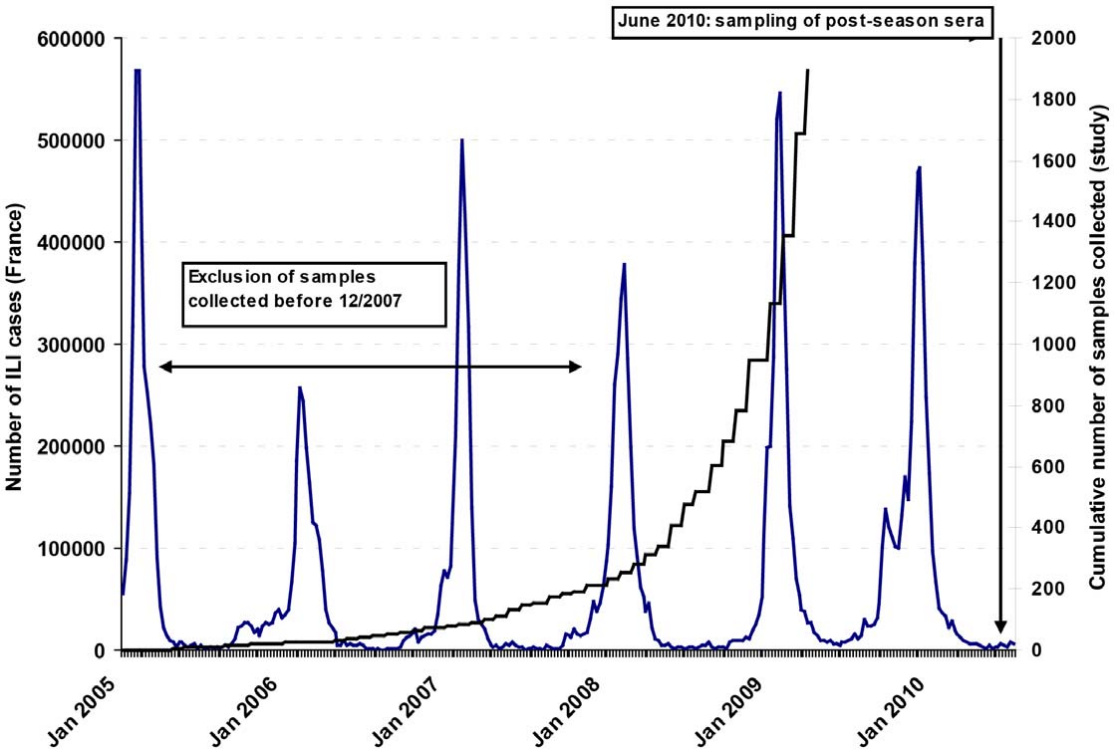
Number of Influenza like illness (ILI) cases seen by the French Sentinel GPs Network (Réseau Sentinnelle, Inserm U707), January 2005–June 2010, and cumulative number of IHA pre-pandemic samples collected for the study, France, 2005–2009.

Between June 21^st^ and July 2^nd^ 2010, 1936 donors were recruited to the study. Compared with samples that were taken before the seasonal influenza epidemic of winter 2007/08 (when a seasonal H1N1 influenza virus antigenically related to A/Brisbane/59/2007 was the predominant circulating strain), samples taken after the start of that season were more likely to be seropositive to the H1N1pdm09 virus than those taken before (crude seroprevalence 6.7% vs. 2.2% p = 0.01). The association remained significant after adjustment for age group and sex (PR 3.1, 95%CI 1.2–8.5). We therefore decided to exclude from the analysis 228 (11.8%) individuals with a pre-pandemic sampling date prior to December 2007 (n = 193) or with an unknown pre-pandemic sampling date (n = 35). Keeping the former individuals would have lead to underestimating the pre-pandemic seroprevalence and hence overestimating the risk of infection. The study design is shown in figure 1 and the characteristics of the 1708 cases eventually included in the analysis are shown in Table 1.

**Table 1:** Prevalence of HI titre ≥1∶40 after the pandemic by exposure variable (weighted) (n = 1708).

Variable		n*	Prevalence (95% CI)	p
**Age-group**	20–29	324	46.80 [29.21, 65.21]	<0.001
	30–39	325	20.88 [14.34, 29.38]	
	40–49	363	16.29 [10.82, 23.77]	
	50–59	363	14.72 [10.57, 20.14]	
	60–70	328	16.23 [11.39, 22.59]	
**H1N1 vaccination status**	Vaccinated	183	73.05 [63.51, 80.85]	<0.001
	Not-vaccinated	1520	18.13 [12.54, 25.48]	
**Titer ≥40 before pandemic wave**	Yes	105	90.18 [80.85, 95.23]	<0.001
	No	1592	18.21 [13.04, 24.84]	
**Sex**	Male	924	26.60 [17.35,38.49]	0.19
	Female	780	19.48 [15.59,24.06]	
**Type of site**	Mobile urban	455	27.08 [17.04, 40.16]	0.15
	Mobile rural	660	20.41 [17.03, 24.25]	
	Fixed	590	15.63 [12.18, 19.82]	
**Influenza – like symptoms**	Yes	74	30.78 [19.11, 45.57]	0.39
	No	1591	22.78 [17.37, 29.27]	
	Don't know	30	32.24 [14.61, 56.95]	

*Totals slightly different from 1708 are explained by a few subjects with missing information for that variable (i.e. 4 subjects with missing information for sex).

### Seroprevalence before the 2009/10 pandemic wave

**Figure 2:**
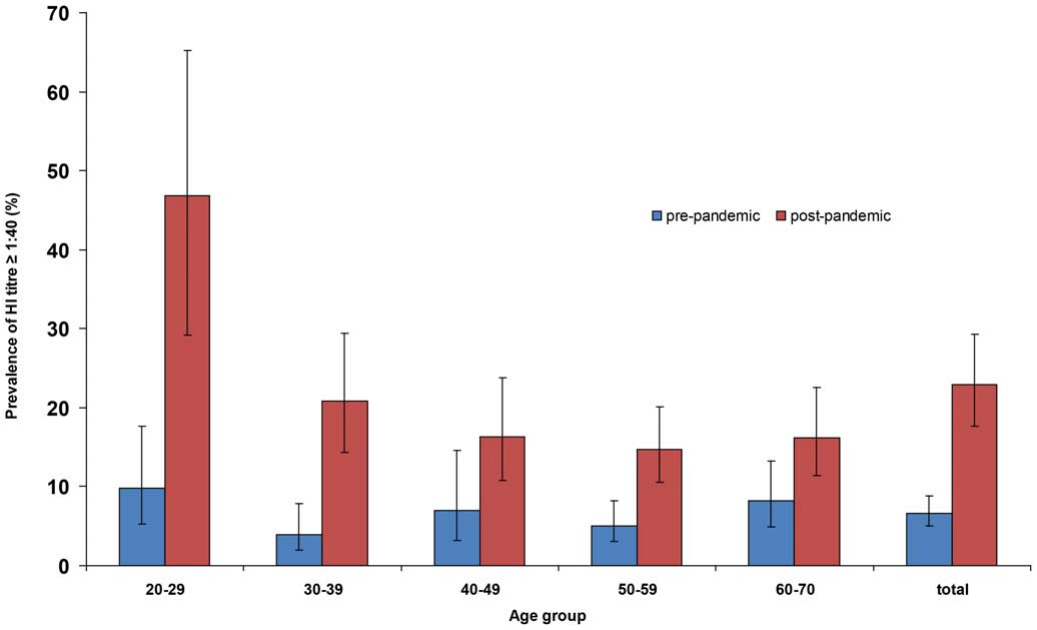
Prevalence of a titre ≥1∶40 before and after the pandemic by age group, France 2010. Seroprevalence before the pandemic was 6.7% (95% CI 5.0–8.9%) overall with no significant differences by age-group. Seroprevalence after the pandemic was 23.0% (95% CI 17.7, 29.3) overall with the 20–29 year age-group having a higher seroprevalence than older groups. The increase in seroprevalence was more marked in younger age-groups.

**Figure 3:**
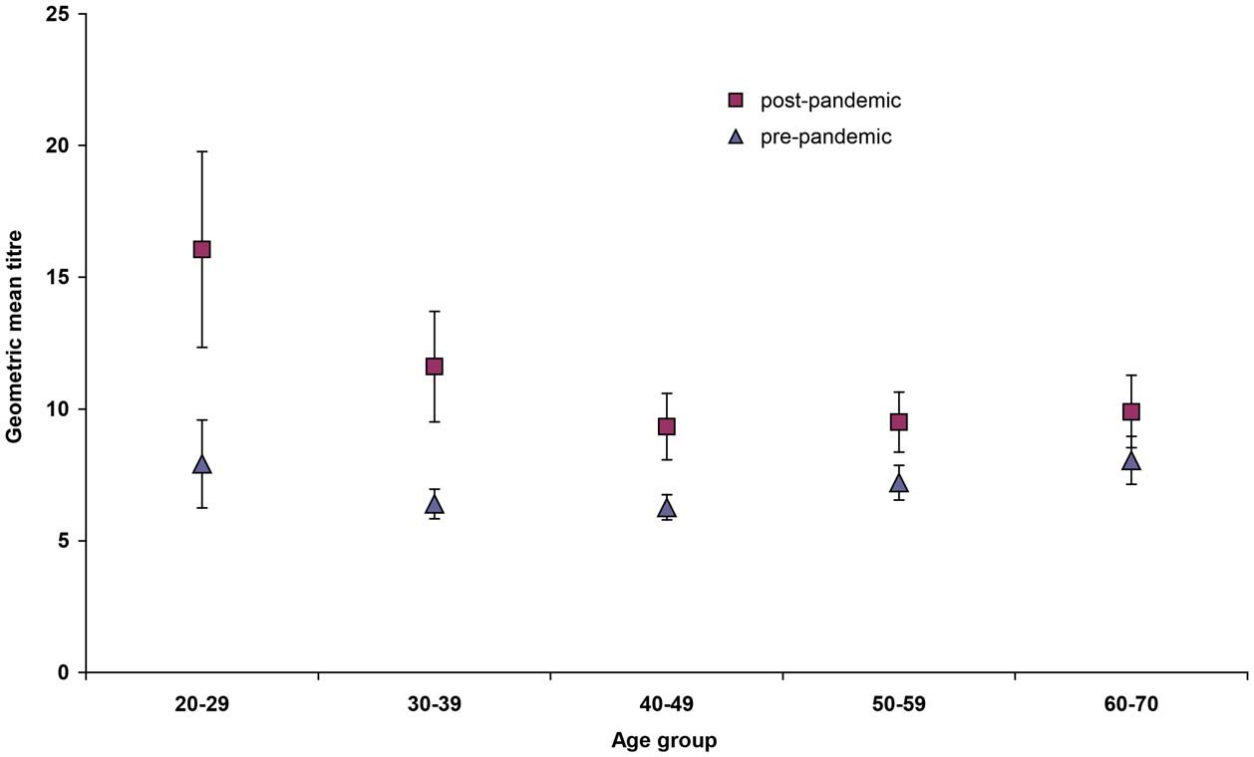
Geometric mean titres before and after the pandemic by age-group, France 2010. In the five age groups, pre-pandemic GMTs ranged from 6.3 to 8.1 with no significant differences by age group. Post-pandemic GMTs showed a significant increase from pre-pandemic results in all age-groups except those aged 60–70, with the increase being more marked in younger groups. Post-pandemic GMTs ranged from 9.3 to 16.1.

Seroprevalence before the pandemic was 6.7% (95% CI 5.0–8.9%) overall with no differences by age-group (p = 0.3) (figure 2), or by sex or region (data not shown). GMTs did not differ significantly by age-group (figure 3). MN assays using the same threshold of 1∶40, gave a seroprevalence of 11.5% (95% CI 7.3–17.7%) overall.

### Seroprevalence after the 2009/10 pandemic wave

**Figure 4:**
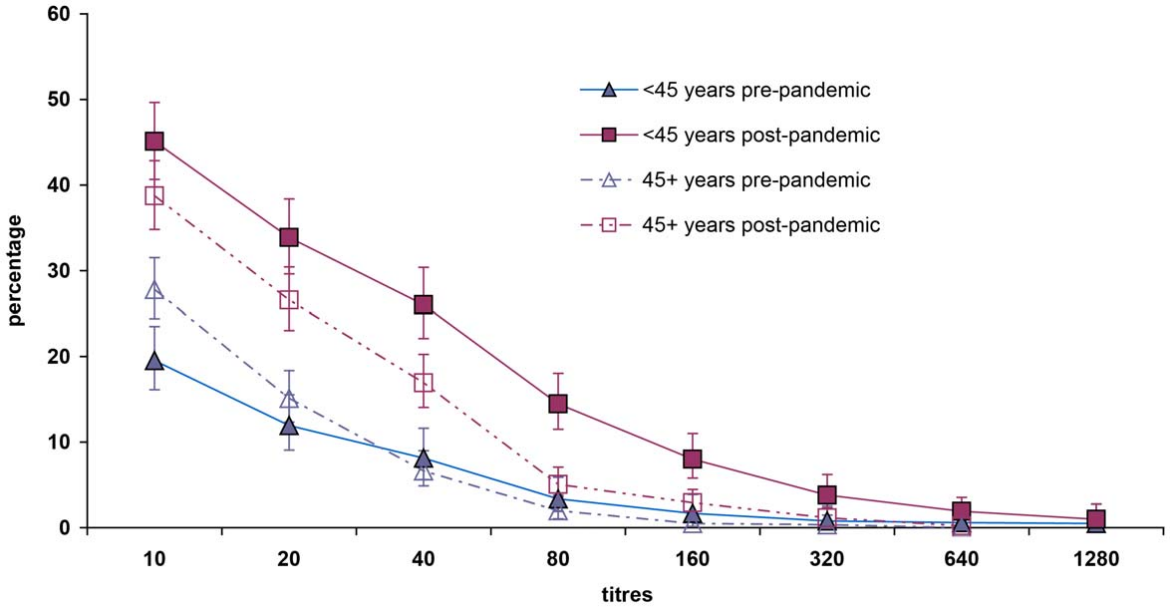
Reverse cumulative distribution curves before and after the pandemic by age group, France 2010. The percentage of the population having a titre equal or superior to a given titre increased between pre-pandemic and post-pandemic samples particularly at lower titres. The increase was more marked in those aged less than 45 years than those 45 and above. Pre-pandemic titres of less than 1∶40 were more common in those aged 45 or more compared with those aged less than 45.

Overall seroprevalence was 23.0% (95% CI 17.7, 29.3) with the 20–29 year age-group having a higher seroprevalence than older groups (p<0.001) (figure 2). GMT's of post-pandemic wave samples were significantly higher than pre-pandemic GMT's in all age-groups except in 60–70 year olds, with the difference becoming less marked with increasing age (figure 3). The percentage of the population having a titre equal or superior to a given titre increased between baseline and post-pandemic wave particularly at lower titres. The increase was more marked in those aged less than 45 years than those aged 45 and above: below 45 years, the proportion of those presenting with a titre below or equal to 40 rose from 8.1% to 26.0%, whereas in those aged 45 years old or more, this proportion rose from 6.6% to 16.9% (figure 4). MN assays using the same threshold of 1∶40, gave a seroprevalence of 22.8% (95% CI 17.4, 29.3%) overall.

On univariable analysis, age-group, vaccination status, seroprevalence before the 2009/10 pandemic wave, sex and type of site were significantly associated with seroprevalence (p≤0.20) (table 1). Seroprevalence was not significantly different in the absence or presence of symptoms, and there were no significant differences by region (data not shown). On multivariable analysis, pre-pandemic seropositivity, vaccination against H1N1pdm09, age 20–29 and type of blood collection site were all found to be significantly associated with being seropositive after the pandemic (table 2). A statistically significant triple interaction was identified between pre-pandemic seropositivity, vaccination and age group. This was used to calculate prevalence ratios for all combinations of these three variables (table 2). Prevalence was significantly higher in 20–29 year olds than 30–70 year olds in the absence of both vaccination and pre-pandemic seropositivity. Vaccination increased post-pandemic seroprevalence in the absence of the pre-pandemic seropositivity and vice versa. Even in the presence of the other variable, both vaccination and pre-pandemic seropostivity resulted in a small increase in prevalence in both age-groups, although this was not statistically significant in the 20–29 age-group.

**Table 2:** Factors associated with HI titre ≥1∶40 after the pandemic wave (multivariable analysis).

Variables			Adjusted PR (95% CI)	p
**Age group 20–29 years compared**	**H1N1 vaccination**	**Titre**		
**to 30–70 years** *				
	Not vaccinated	Titre <1∶40 pre-pandemic	4.99 [2.65, 9.38]	<0.001
	Vaccinated	Titre <1∶40 pre-pandemic	1.33 [0.91, 1.94]	0.14
	Not vaccinated	Titre ≥1∶40 pre-pandemic	1.07 [0.87, 1.32]	0.50
	Vaccinated	Titre ≥1∶40 pre-pandemic	1.12 [0.85, 1.48]	0.41
**H1N1 Vaccination compared**	**Age-group**	**Titre**		
**to no vaccination** *				
	20–29	Titre <1∶40 pre-pandemic	2.57 [1.53, 4.32]	<0.001
	20–29	Titre ≥1∶40 pre-pandemic	1.30 [0.94, 1.80]	0.11
	30–70	Titre <1∶40 pre-pandemic	9.63 [6.49, 14.30]	<0.001
	30–70	Titre ≥1∶40 pre-pandemic	1.25 [1.02, 1.53]	0.03
**HI Titer ≥1∶40 before pandemic**	**Age-group**	**H1N1 Vaccination**		
**compared to HI titer <1∶40** *				
	20–29	Not vaccinated	2.51 [1.41, 4.47]	0.002
	20–29	Vaccinated	1.27 [0.86, 1.87]	0.23
	30–70	Not vaccinated	11.65 [7.61, 17.82]	<0.001
	30–70	Vaccinated	1.51 [1.25, 1.82]	<0.001
**Sex** - Male compared to female			1.33 [0.95, 1.87]	0.10
**Type of site -** Mobile urban compared to fixed			1.71 [1.22, 2,38]	0.002
**Type of site -** Mobile rural compared to fixed			1.36 [1.09, 1.71]	0.007
**ILI symptoms** compared to no ILI symptoms			1.31 [0.86, 2.00]	0.21

* Prevalence ratios calculated using variables forming the statistical triple interaction identified (age group, H1N1 vaccination and pre-pandemic titre ≥1∶40).

### Seroconversion

The proportion of the population seroconverting overall was 16.0% (95% CI 11.2, 22.3). In those who were not seropositive before the pandemic, 65.4% (95% CI 55.0, 74.5) of those vaccinated seroconverted. The proportion seroconverting due to infection was 12.2% (95% CI 6.9, 20.5), with a significantly higher proportion in those aged 20–29 compared with other age-groups (table 3). Among those seroconverting through infection, 89.6% (95% CI 76.7, 95.8%) reported no influenza-like symptoms during the 2009/10 wave. On multivariable analysis, age 20–29 years, mobile blood collection sites and having had symptoms were all significantly associated with seroconversion attributable to infection.

**Table 3:** Factors associated with seroconversion in those without pre-pandemic titre ≥1∶40 or vaccination against H1N1.

Variable		n	Prevalence %	P	Adjusted Prevalence	p
			(95% CI)		Ratio (95% CI)	
**Age-group (years)**	20–29	273	40.36 [19.32, 65.67]	<0.001	6.17 [3.39, 11.24]	<0.001
	30–39	265	9.98 [4.51, 20.66]		Ref 30–70 years	
	40–49	310	3.30 [1.64, 6.52]			
	50–59	303	4.95 [2.71, 8.89]			
	60–70	272	3.29 [1.75, 6.10]			
**Sex**	Male	764	15.74 [6.50, 33.45]	0.22	1.47 [0.79, 2.73]	0.22
	Female	659	8.67 [5.95, 12.47]		Ref	
**Type of site**	Mobile urban	385	18.03 [8.29, 34.87]	0.02	3.25 [1.72, 6.13]	<0.001
	Mobile rural	542	7.30 [5.15, 10.26]		1.88 [1.03, 3.43]	0.04
	Fixed	497	4.62 [2.67, 7.90]		Ref	
**ILI symptoms**	Yes	61	19.99 [10.16, 35.57]	0.38	1.88 [1.00, 3.53]	0.05
	No	1330	11.94 [6.32, 21.41]		Ref no or don't know	
	Don't know	26	21.06 [6.25, 51.61]			

## Discussion

We found seroprevalence after the 2009/10 pandemic wave to be 23.0% overall, with higher seroprevalences in younger age-groups. Seroprevalence was 6.7% before the pandemic with no significant differences by age-group. We estimated the proportion of the population having seroconverted as a result of infection to be 12.2%, with a significantly higher proportion in the population aged 20–29. Our results are generally consistent with those published from other countries although comparisons between studies are limited because of the use of different age-groups, techniques, thresholds etc [5].

Most estimates of the proportion of the population infected have been extrapolated from seroprevalence data obtained from convenience samples of different subjects before and after the pandemic [4], [16], [17] [18]–[20], one of which also incorporated clinical surveillance and data on seroconversion intervals collected from pandemic influenza cases in their estimates [21]. A few studies have estimated seroconversion before and after the first pandemic wave using the same subjects, but again different methodologies limit direct comparison. Chen et al demonstrated seroconversion in 13.5% (95% CI 11.2,16.2) of participants providing paired samples in an unvaccinated population in Singapore using HI assays [6]. In Hong Kong, Wu et al performed MN assays on paired sera from 324 blood donors as part of a larger serial cross-sectional serological study, reporting lower seroconversion rates in all age groups aged 20–60 years, having excluded those with a pre-pandemic MN titre of <1∶10 (for example 5.3% (95%CI 1.7, 11.9) in 20–29 year olds) [8]. In a community-based paired serological study again using MN assays in Hong Kong, Riley et al reported seroconversion rates of 8.9% (95% CI 5.3, 14.7) in 20–39 year olds and 5.3% (3.5, 8.0) in 40–59 year olds [7].

Our estimate of seroconversion due to infection is likely to be an underestimate as we excluded those with a pre-pandemic titre of ≥1∶40 and those vaccinated for these analyses. However, a 4-fold increase in antibodies as a result of infection could have occurred in those with a pre-pandemic titre of ≥1∶40. Subsequent analysis indicated that of the small proportion who were seropositive before the pandemic and unvaccinated, 3.8% mounted a 4-fold increase in titre. Moreover, we assumed that seroconversion in the presence of vaccination was due to vaccination, although alternative causes could be infection prior to vaccination or infection in the context of vaccine failure. Lastly infection may have resulted in a less than 4-fold increase in titre.

The finding that nearly 90% of those seroconverting as the result of infection reported not having had an influenza-like illness was surprising as the proportion of asymptomatic or mildly symptomatic infections in seasonal influenza is usually in the range of 40–60% [22]. Other authors have found the proportion of H1N1pdm09 infections that were asymptomatic or pauci-symptomatic to be 27%–82% [6], [16], [23] [7], [24]. However, our definition of influenza-like illness likely excluded mild presentations of disease and problems with recall as our study was carried out six months after the pandemic cannot be excluded.

Our pre-pandemic seroprevalence results are generally consistent with other studies in this age group (20–70 years) [4], [8], [16], [17], [20], [25]–[28]. Higher pre-pandemic seroprevalences have been observed in older age-groups, particularly in western countries, but almost always in age-groups beyond the age of 70 [4], [27], [29]–[31]. One French study has reported very different results using the HI method and a threshold titre of 1∶40, with pre-pandemic seroprevalences of between 43% and 69% in those aged 25–100 years based on a convenience sample of serum stored from hospital patients in the Marseilles region [19]. However, these authors also report the findings of a parallel national study of pregnant women carried out during the pandemic with an estimated baseline seroprevalence at the start of the pandemic of 2.4% (threshold titre 1∶80). The most likely explanation for these differences seems to be different sampling strategies, source populations and laboratory methods. In our study, pre-pandemic seroprevalence was higher in samples taken after the start of the winter influenza season 2007–2008, an observation we believe has not been previously reported. Contact with the seasonal A/Brisbane/59/2007-like viruses, which were the predominant viruses circulating that season, may have enhanced homosubtypic cross-reactive immunity [32].

Our post-pandemic seroprevalence results are also compatible with most other studies, although higher post-pandemic seroprevalences are reported from countries and regions with better H1N1pdm09 vaccine uptake [18], [33], [34]. Post-pandemic seroprevalence was higher in younger age-groups, the vaccinated and in those who were seropositive before the pandemic. Seventy-one percent of those reporting H1N1pdm09 vaccination had a level of antibodies usually considered to be protective in June 2010, consistent with published estimates of vaccine effectiveness [35]. Since our study was carried out six months after the vaccination campaign, our result may underestimate vaccine associated protection because of waning immunity [36]. However, a meta-analysis has been recently performed by WHO including all published and unpublished sero-studies for the 2009 influenza pandemic virus, including ours. It has actually shown a decrease in seroprevalence with time, in the 40 weeks interval between sera collection and peak in influenza activity, however this decrease was statistically non significant. Ninety percent of those who were seropositive before the pandemic were seropositive afterwards. Analysis of cases where the titre dropped below 1∶40 revealed changes around the threshold (most commonly from a titre of 1∶40 to a titre of 1∶20) probably due to variations within the assays. In multivariable analysis, three variables (age-group, vaccination and pre-pandemic seropositivity) were strongly associated with seroprevalence and all found to modify the effect of each other (table 2). Younger age-group was only associated with seropositivity after the 2009/10 pandemic wave in the absence of vaccination and/or pre-pandemic seropositivity.

A significant association was also found with type of blood collection site, with levels of seropositivity and seroconversion being significantly higher in populations attending mobile sites compared with fixed sites. This difference may reflect factors influencing exposure, such as mobility or socioeconomic differences.

Our seroprevalence results are rather different from French modelling estimates based on general population surveillance data (adjusted for the estimated proportion of asymptomatic infections [22], [37] and healthcare seeking behaviour [38]), which found the proportion of the population aged 19–64 years immune to H1N1pdm09 before the 2009/10 pandemic wave to be 36% [38]. The disparity between the model and serological estimates raises questions regarding which estimates reflect the real levels of protection. Models may under or overestimate prevalence as they use a series of assumptions. But serological assays are likely to underestimate protection as they cannot quantify cross-reactive immunity with closely related H1N1 viruses, nor the cross-reactive cellular responses acquired through past infections [39], [40]. As post-pandemic wave samples were collected 6 months after the epidemic, a decline in detectable antibody titres over time, cannot be ruled out [36]. Furthermore, the threshold of an HI titre of ≥1∶40 as an indicator of seroprotection can be challenged [41], and has not been specifically validated for H1N1 2009 or in all age-groups. MN assays detect a broader range of antibodies than HI but there is no established correlate of protection. If the presence of any antibody detected by MN were to be considered protective, seroprevalence after the 2009/10 pandemic wave would be as much as 46% (95% CI 40, 52).

Our choice of blood donors to represent the general adult population warrants consideration. Certain groups are excluded from blood donation, mainly pregnant women, people presenting with some chronic illnesses, with blood borne infections or with risk factors for their acquisition. However we believe that this should have a very limited impact on our estimations as those characteristics are likely to be poorly correlated with the pre-pandemic serostatus and the risk of infection during the pandemic wave. The fact that the vaccination coverage of our sample (8.9%) tended to be slightly higher that the one measured in the general population of the same age, through the national H1N1pdm09 vaccination campaign database (6%) is not in favour of a bias toward selection of healthier subjects [3]. Similarly, we did not formally monitor the number of donors refusing to participate, but we do not consider selection bias to be a significant problem as it seems unlikely that participation to the study would be linked to the outcomes studied. A further consideration is that the blood bank provided plasma specimens, whilst the reference techniques described by the World Health Organization for serological assays are based on sera. No head to head comparisons between sera and plasma HI titres have been published, but in our experience there are no significant differences between HI titres when using plasma as compared to sera. Considering however that HI reactions are sometimes incomplete with plasma, which may putatively have an impact of HI values, we selected the most appropriate red blood cells, and added an additional reading step after 60 min at +4°C. With this technical adaptation, the readings were consistent, and specimens remaining difficult to read (partial HI) were retested. Finally, an important weakness of our study linked to our choice of study population is that we were not able to assess seroprevalence and seroconversion in children, the population shown by others to have been most infected by the virus, because of the population studied. Another serological study looking at the prevalence of antibodies to several infections including H1N1pdm09 in a random selection of individuals age 6–29 attending private laboratories in France is currently being analysed.

### Conclusions

Our methodology has enabled us to study seroprevalence in the French mainland population aged 20–70 years by collecting data from the same individuals before and after the 2009/10 pandemic wave, and to examine the effect of vaccination and pre-pandemic seropositivity on post-pandemic wave seroprevalence. We were also able to provide direct estimates of the proportion of the population seroconverting as a result of infection. We found a high proportion of pauci- or asymptomatic infections. Pre-pandemic seroprotection was higher in samples taken after the winter 2007/2008 influenza season suggesting that the seasonal H1N1 virus circulating in that season may have contributed to pre-existing seroprotection.

Our results indicate that almost 80% of the French mainland population aged between 20–70 years did not have a protective level of antibodies at the start of the 2010/2011 winter influenza season. Whether this implies that this proportion of the population was still susceptible to influenza H1N1pdm09 virus is still unclear, given that measurement of antibody titres alone cannot fully quantify immunity and that models suggested higher seroprevalences. At the time, the season's vaccine recommendations were to offer vaccine to traditional at-risk groups for seasonal influenza (those aged 65 and over and those with certain chronic diseases). In view of our estimates, and reports from the southern hemisphere and England and Wales of high proportions of severe influenza cases in 2010 [42], taken with the poor uptake of vaccine by the general population during the pandemic, the decision was made to extend the season's vaccine recommendations to additional risk groups particularly affected during the pandemic (pregnant women and obese people) [43].

## Supporting Information

Appendix S1 Supplementary information on calculation and calibration of sampling weights and choice of the Poisson regression model.
(DOC)
