# Supplementary material for: Incidence of H1N1 2009 Virus Infection through the Analysis of Paired Plasma Specimens among Blood Donors, France
Source: PLoS One. 2012 Mar 22;7(3):e33056. doi: 10.1371/journal.pone.0033056 (PMC3310844; doi:10.1371/journal.pone.0033056)
Supplement: Appendix S1 — Supplementary information on calculation and calibration of sampling weights and choice of the Poisson regression model. (DOC) [file pone.0033056.s001.doc]

**e-Appendix – S1**

**Calculation of sampling weights**

A two-stage random sampling design, stratified by French blood service regions (EFS regions), type of site and age-group was performed.

At the first stage, the sampling frame consisted in 339 blood collection sites planned for study period. This sampling frame was stratified by 14 mainland EFS regions and type of blood collection site (fixed, mobile urban, mobile rural).

In each stratum, sites were selected using the ordered systematic sampling with unequal probabilities (Tillé, p124-127), proportional to regular donor activity in June 2009. For each stratum *h* (*h=1,…,H*), if we note *X_kh_* the regular donor activity of the site *k*; N_h_ the number of sites in the sampling frame and n_h_ the number of sites drawn, then the inclusion probability of the site *k* in the stratum *h* is :

$$\pi_{kh}^{(1)}=\frac{n_{h}\times X_{kh}}{\sum_{i=1}^{N_{h}} X_{kh}}$$

At the second stage, donors were randomly recruited from a sampling that can be view reasonably as a simple random sampling. If we note *m_k_* the number of donors recruited in the site *k* and *M_k_* the number of donors in the site *k*, the inclusion probability for a donor in the site *k* is equal to:

$\pi_{k}^{(2)}=\frac{m_{k}}{M_{k}}$.

For each donor *i* recruited in the site *k* belonging to the stratum *h*, a sampling weight is calculated as:

$$w_{i}=\frac{1}{\pi_{kh}^{(1)}\times\pi_{k}^{(2)}}$$

**Calibration**

Sampling weights were modified by post-stratification taking into account census data. More precisely, a coefficient was calculated in each post-stratum (defined accordingly to sex and five age groups). For each post-stratum, the coefficient was calculated as the real number of individuals in the population of interest divided by the estimated number of individuals in the population of interest.

Then, new sampling weights were calculated as the product of the sampling weights defined in the previous section and the coefficients. These new sampling weights were used to obtain the final estimates (prevalences, prevalence ratio etc.).

The estimated post-stratified age-sex distribution and the age-sex census distribution are thus strictly identical as shown in the following table.

| **Variable** |  | **French population (2008 census)** | **Donors population (2010 data)** | **Age-sex post-stratified estimates from the sample** |
| --- | --- | --- | --- | --- |
| **Age group** |  |  |  |  |
|  | 20-29 | 19.8% | 23.9% | 19.8% |
|  | 30-39 | 21.1% | 18.7% | 21.1% |
|  | 40-49 | 22,0% | 24.8% | 22,0% |
|  | 50-59 | 21,1% | 21.9% | 21,1% |
|  | 60-70 | 16.0% | 10.6% | 16.0% |
| **Sex** |  |  |  |  |
|  | Male | 49.2% | 49.5% | 49.2% |
|  | Female | 50.8% | 50.5% | 50.8% |
| **Region** |  |  |  |  |
|  | Alpes Méditerranée | 8.4% | 7.2% | 6.9% |
|  | Alsace | 3.0% | 4.0% | 4.8% |
|  | Aquitaine Limousin | 6.3% | 7.1% | 6.2% |
|  | Auvergne Loire | 3.5% | 4.2% | 4.9% |
|  | Bourgogne FC | 4.5% | 5.2% | 7.8% |
|  | Bretagne | 5.1% | 6.3% | 5.2% |
|  | Centre Atlantique | 5.6% | 7.3% | 5.9% |
|  | Ile-de-France | 18.8% | 11.0% | 22.0% |
|  | Lorraine Champagne | 5.9% | 5.9% | 4.5% |
|  | Nord de France | 9.5% | 10.4% | 8.1% |
|  | Normandie | 5.3% | 5.5% | 5.3% |
|  | Pays de la Loire | 5.7% | 5.4% | 5.3% |
|  | Pyrénées Méditerranée | 8.7% | 9.5% | 6.3% |
|  | Rhône Alpes | 9.8% | 10.9% | 6.5% |

**Choice of the Poisson regression model**

The main reason to consider as log-Poisson regression model is based on the fact that a logistic regression is not adapted to our data.

1) As the survey design is a cross-sectional design, we are interested in prevalence ratios and not in odds ratios that are just an approximation of prevalence ratios.

2) It is known that an odds ratio well approximates a prevalence ratio if and only if the prevalences among groups are not so different. This is not the case in our study.

3) We chose a logarithmic link between the outcome of interest and covariables to make sure that the exponential of the regression coefficients are equal to the prevalence ratio.

4) As the outcome of interest is a binary outcome, we chose a discrete distribution. It is established that the binomial distribution causes some convergence problems when the number of covariables increase (Zou, 2004). The use of a Poisson distribution decreases the risk of convergence problems, even if other problems can arise with the log-Poisson model (Blizzard & Hosmer, 2006).

As mentioned in the article, the multivariable model was build by stepwise selection, including the sampling design. Then some interaction terms (only those which are relevant from an epidemiological point of view) were added and tested for significance.

**References**

Blizzard L, Hosmer DW (2006) Parameter estimation and goodness-of-fit in log binomial regression. Biometrical Journal, 1, 5-22.

Tillé Y Sampling algorithms. Springer Series in Statistics.2006

Zou G (2004) A modified Poisson regression approach to prospective studies with binary data. Am J Epidemiol; 159, 702-706.
